# Successful open surgical treatment for persistent type II endoleak following thoracic endovascular aortic repair: a case report

**DOI:** 10.1186/s13019-024-02843-3

**Published:** 2024-07-03

**Authors:** June Lee, Do Yeon Kim, Hyun Ah Lim, Seok Beom Hong, Yong Han Kim, Hwan Wook Kim

**Affiliations:** 1 Department of Thoracic and Cardiovascular Surgery, Uijeongbu Eulji Medical Center, Eulji University, Uijeongbu-si, Republic of Korea; 2 Department of Thoracic and Cardiovascular Surgery, Seoul St. Mary’s Hospital, College of Medicine, The Catholic University of Korea, 222 Banpo-daero, Seocho-gu, Seoul, 06591 Republic of Korea

**Keywords:** Endoleak, Endovascular aneurysm repair, Thoracic aorta

## Abstract

**Background:**

The occurrence of type II endoleaks after endovascular repair of aortic aneurysm has gradually gained increasing attention. We present a case of a patient with an expanding aneurysm after thoracic endovascular aortic repair (TEVAR) for a type II endoleak, in which successful direct ligations of the intercostal artery were performed using a sac incision without cardiopulmonary bypass (CPB) or graft replacement.

**Case presentation:**

A 62-year-old male patient, previously treated with TEVAR for a descending thoracic aortic aneurysm, presented with ongoing chest discomfort. Based on the diagnosis of a growing aneurysm and type II endoleak, the patient was prepared for CPB and aortic cross-clamping, as a precautions against the possibility of a type I endoleak. A longitudinal opening of the thoracic aortic aneurysm sac was performed following left thoracotomy. Visual confirmation identified the T5 level intercostal artery as the source of the endoleak, and after confirming the absence of a type I endoleak, multiple ligations were applied to the intercostal artery. Follow-up computed tomography confirmed the absence of endoleaks or sac growth.

**Conclusion:**

In a case involving TEVAR for a thoracic aortic aneurysm, open suture ligations were used to treat type II endoleaks without having to resort to CPB, resulting in successful outcomes.

## Background

After endovascular repair of an aortic aneurysm, type II endoleaks emerge as a predominant complication. These may originate from retrograde flow through collateral vessels into the aneurysm sac. Type II endoleaks are reported to occur in more than 10% of patients undergoing endovascular abdominal aneurysm repair (EVAR) and represent approximately half of all endoleaks [1]. Abdominal aortic aneurysms can rupture due to type II endoleaks in approximately 1% of cases; among these, about one-third occur in the absence of aneurysm sac expansion. According to Seike et al. [2], there is an association between persistent type II endoleaks and increased incidence of abdominal aortic aneurysm sac enlargement, the need for reintervention, rupture, and increased mortality related to aortic aneurysms.

In the context of thoracic endovascular aortic repair (TEVAR), at a median follow-up of approximately 29.5 months, 8.7% of patients exhibit type II endoleaks, of which 43.3% originate from the intercostal or bronchial vessels [3]. In particular, within this subset of patients with type II endoleaks, a mortality rate of 6.7% due to associated ruptures was observed, which highlights the importance of avoiding the formation of type II endoleaks. The incidence of type II endoleaks, depending on the institution performing endovascular stent graft insertion in the thoracic aorta or the extent of the disease, has been reported to be as high as approximately 12~29% [4, 5], warranting attention.

In this paper, we report a rare case of a patient presenting persistent type II endoleaks and the development of an expanding aneurysm post-TEVAR. The patient underwent a left lateral thoracotomy and a sac incision of the thoracic aortic aneurysm, which resulted in the successful ligation of the intercostal artery responsible for the type II endoleak.

## Case presentation

**Fig. 1:**
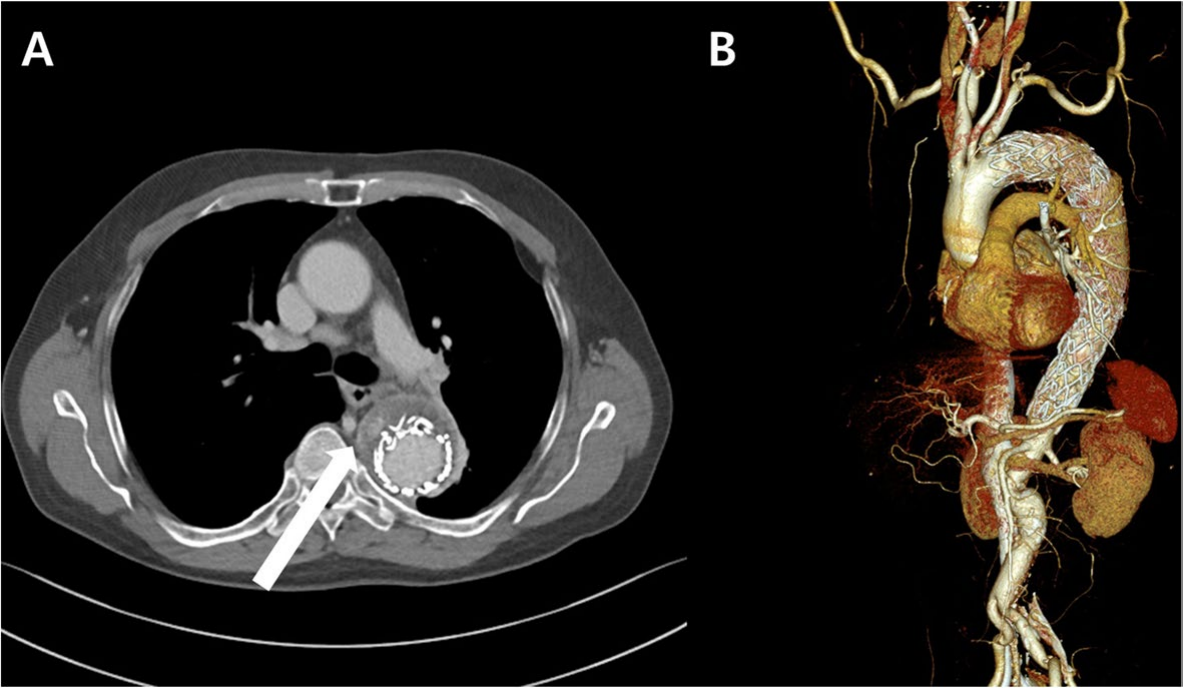
Preoperative CT angiogram. **a** Descending thoracic aortic aneurysm (maximal dimension of the aneurysm = 5.9 cm) with a type II endoleak (white arrow) in the delayed phase. **b** Post-TEVAR state showing multiple stent grafts from zone 3 to 5 of the aorta. CT: computed tomography; TEVAR: thoracic endovascular aortic repair

A 62-year-old male patient who had previously undergone TEVAR for a descending thoracic aortic aneurysm presented ongoing chest discomfort. The patient was referred to the Department of Thoracic Cardiovascular Surgery at our institution. The patient had a history of underlying medical conditions, including diabetes, hypertension, and smoking. Additionally, he had undergone open repair for an abdominal aortic aneurysm nine years prior. Approximately four years prior, he developed an intramural hematoma along with a descending thoracic aortic aneurysm (5.7 cm), which led to his zone 3 [6] TEVAR procedure using a Medtronic Valiant thoracic endograft (Medtronic Parkway Minneapolis, MN, USA) (38×38×200 mm). Three years later, due to the expansion of an aortic aneurysmal sac, he underwent an additional TEVAR procedure with Valiant thoracic endografts (proximal 42×42×200 mm, distal 38×38×200 mm) under suspicion of type IA or B endoleak. However, due to the continuous growth of the descending thoracic aortic aneurysm (5.9 cm) and the suspicion of a type II endoleak (Fig. 1), the decision was made to proceed with open repair. The proposed open surgical repair plan involved performing an open thoracotomy, followed by an incision of the enlarged aneurysm sac. Subsequently, in the case of type IA or B endoleak, the plan was to perform aortic graft replacement using descending aorta cross clamps and cardiopulmonary bypass (CPB). In cases where a type II endoleak is detected, it was planned to directly ligate the intercostal arteries suspected of causing the endoleak.

**Fig. 2:**
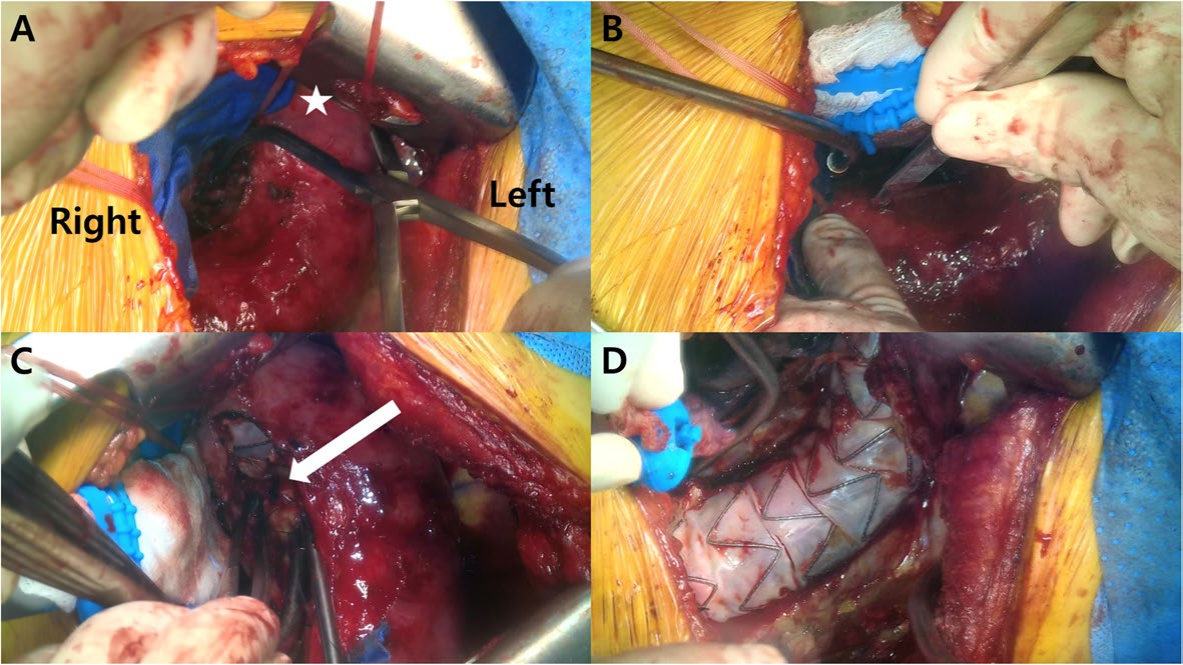
Intraoperative findings. **a** Testing the readiness to apply the cross clamp by encircling the umbilical tape around the proximal descending aorta (white star). **b** Deliberate incision into the aortic aneurysm sac to assess bleeding patterns in preparation for aortic cross clamp application when a type I endoleak is suspected. **c** Ligation of the intercostal artery (white arrow) with 4-0 prolene suture performed after confirming the absence of a type I endoleak and presence of only a type II endoleak. **d** Confirmed absence of an endoleak

**Fig. 3:**
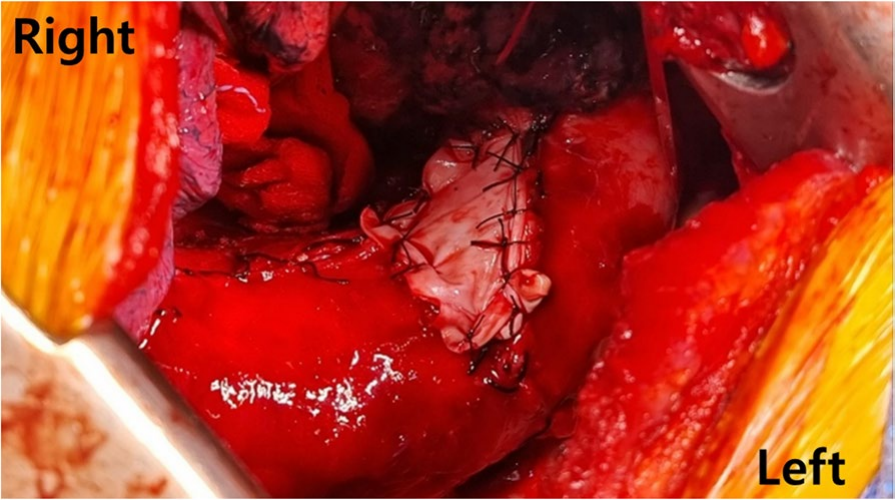
The aortic aneurysm sac closure achieved with bovine pericardium

While employing one-lung ventilation, we placed the patient in the right lateral decubitus position and provided access to the left femoral artery and vein to prepare for a potential CPB. We performed a left thoracotomy through the 5th intercostal space and delicately dissected the severe adhesions around the lung and the descending aorta. To prepare for a possible type I endoleak, we readied aortic cross-clamps to be positioned in both the proximal and distal sections of the descending thoracic aorta after circumferential dissection and looping with umbilical tapes (Fig. 2A). The aneurysm sac was opened slowly and carefully (Fig. 2B). Following longitudinal opening (approximately from T4 to T8 levels) of the chronic descending aortic aneurysm, we detected and cleared the old hematoma and debris, noting ongoing bleeding. After rinsing with saline, a visual check confirmed the absence of type IA or B endoleaks. However, we observed bleeding from a single intercostal artery at the T5 level, suggesting an association with a type II endoleak. As a result, multiple ligations of the intercostal artery were performed using pledgeted prolene 3-0 sutures (Fig. 2C). After completing surgery without the need for CPB, we performed aneurysmorrhaphy using a bovine pericardial patch in the sac of the aneurysm (Fig. 3). The entire surgical procedure lasted 3 h and 36 min.

**Fig. 4:**
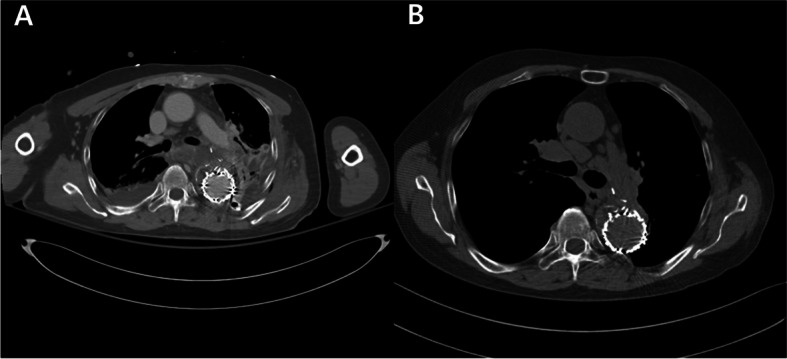
Postoperative CT findings. **a** Complete absence of any remaining endoleak and a well-repaired descending thoracic aorta before discharge. **b** Non-enhanced CT imaging at 6-month outpatient follow-up showing no size changes in the descending aorta. CT: computed tomography

The patient was extubated on the day of surgery. On day two of postoperative care, the computed tomography (CT) confirmed the absence of any abnormalities at the surgical site and ruled out the presence of an endoleak (Fig. 4A). The chest tube was removed on postoperative day 8. The patient was discharged on postoperative day 11. It was decided to continue clopidogrel 75mg qd upon discharge for outpatient follow-up. For approximately six months, the patient underwent follow-up appointments at the outpatient department, with no symptoms observed during these visits (Fig. 4B).

## Discussion

When it comes to EVAR, previous claims have indicated that the presence or absence of a type II endoleak that persists for 6 months or more had no effect on survival [7, 8]. Furthermore, it has been suggested that patients who undergo secondary interventions for type II endoleaks did not show enhanced survival compared to those without such interventions [8]. These factors minimized the importance of type II endoleaks. However, the significance of type II endoleaks is increasing and is supported by research findings indicating their growing impact on aortic rupture and mortality [1, 2].

As per a clinical practice guideline, after EVAR, a diameter increase of more than 1 cm in the aneurysm sac over a period of one year is considered a significant enlargement, prompting Class IIa and Level C recommendations for re-intervention to address the type II endoleaks [9]. The primary approach to managing type II endoleaks involves embolization strategies utilizing techniques such as transarterial methods and direct sac puncture (including translumbar, transabdominal, and transcaval approaches) [10]. Internal or external vascular ligation has been frequently reported after EVAR to address type II endoleaks [11]. The primary contributors to type II endoleaks post-EVAR, such as the inferior mesenteric or lumbar arteries, are often addressed by ligation. Internal ligation involves an incision and suture ligation within the aneurysm sac, whereas external access allows easier targeting using clips. Opting for an entire-sac graft replacement increases surgery time and risks, favoring ligation as a less invasive option.

However, when it comes to TEVAR, several studies [12–14] indicate that when assessing the results of TEVAR for various conditions, such as aortic dissection and aneurysms, some studied have not explicitly identified type II endoleaks or have deemed them less significant. Even in one study [15] mentioning type II endoleaks, the majority of surgeons opted for conservative care, with approximately one case reported to have received coil embolization. To our knowledge, there have been no instances of type II endoleaks that arise from the intercostal arteries after TEVAR in the thoracic aorta using ligation.

We successfully completed the procedure after a thoracotomy without CPB. We precisely identified the intercostal artery responsible for the type II endoleak post-sac incision and verified the cessation of bleeding through internal ligation. Targeting type II endoleaks in the descending aorta at the thoracic level is an exceptionally rare technique. We are eager to share this approach, as it seems promising and viable.

However, our study highlights several cautionary aspects of this surgical method. It is difficult to locate and tie off the intercostal artery outside the aneurysm sac. Severe adhesions around the thoracic aortic aneurysm can add complexity, casting doubt on whether the ligated intercostal artery identified on the preoperative CT scan is the actual cause. Opening the aneurysmal sac for direct visual inspection is the most precise approach. In addition, the presence of tiny, unnoticed type I endoleaks prior to surgery may have contributed to the incomplete resolution in this patient.

Similarly, given the potential of a type I endoleak, it is wise to prepare the CPB. It is recommended to ensure easy access to the left femoral artery and vein and prepare to position the aortic cross-clamp at the proximal and distal ends of the descending aorta, where surgical intervention is planned. It is crucial to perform a thorough check when opening the aneurysmal sac to ensure the complete elimination of a type I endoleak. If significant and marked bleeding is observed after cutting the aortic aneurysm, this indicates a severe situation, and a type I endoleak should be suspected. Therefore, our strategy included immediate initiation of heparinization, CPB, and prompt application of an aortic cross-clamp. The method used in this case is not the same, but it is anticipated that applying banding to the proximal or distal descending aorta to treat type I endoleak would be a very effective technique. Hence, a cautious and intentional approach to opening an aortic aneurysm aims to closely observe the bleeding pattern.

In the case described here, the prolonged surgical duration was attributed to severe adhesions that required extensive dissection, delaying the preparation for applying cross-clamps at both the proximal and distal segments of the descending thoracic aortic aneurysm. Additionally, upon opening the aneurysm sac, needling was challenging because of the limited space between the aneurysm sac and the endovascular stent graft, as we wanted to ligate the internal identified intercostal artery.

## Conclusions

Based on our experience, the treatment of type II endoleaks by direct suture ligation has been successful, is feasible, and is not intractable. With this report we wanted to share our experience with internal ligation treatment via a thoracotomy and a sac opening to highlight the advantages of simplified surgical methods in the absence of aortic graft replacement or CPB.

## Data Availability

No datasets were generated or analysed during the current study.

## Abbreviations

EVAR: Endovascular abdominal aneurysm repair
TEVAR: Thoracic endovascular aortic repair
CPB: Cardiopulmonary bypass
CT: Computed tomography
